# Dual Function Molecules and Processes in Cell Fate Decision: A Preface to the Special Issue

**DOI:** 10.3390/ijms21249601

**Published:** 2020-12-16

**Authors:** Sonia Emanuele, Michela Giuliano

**Affiliations:** 1 Department of Biomedicine, Neurosciences and Advanced Diagnostics (BIND), Biochemistry Building, University of Palermo, Via del Vespro 129, 90127 Palermo, Italy; 2 Department of Biological, Chemical and Pharmaceutical Sciences and Technologies (STEBICEF), Laboratory of Biochemistry, University of Palermo, Via del Vespro 129, 90127 Palermo, Italy

A lot of water has passed under the bridge since 1999, when C.J. Jeffery stated in a pioneering review that “the idea of one gene-one protein-one function has become too simple” [1], and presented to the scientific community the concept of “moonlighting proteins”, indicating with this term proteins that can perform different, often unrelated, functions in the cells.

Since then, examples of moonlighting proteins have considerably increased, and with them the terminology to explain the functional heterogeneity and the diverse behavior. Since then, it has been realized that even simply cataloguing proteins was more than complex. For example, in cancer research, distinguishing oncogenes from tumor suppressors seemed initially easy. However, over time ambiguous genes in cancer were identified, which play opposite roles depending on gene mutation and environmental conditions. One example for all is p53, which can turn from tumor suppressor to oncogene due to a switch from loss of native function to gain of toxic function [2,3]. More recently, the term “neomorphic” was coined to indicate a mutation that confers novel molecular functions [4].

Opening this Pandora’s box has overcome boundaries that were once unthinkable. Not only can single molecules or enzymes modify their role due to gene mutation or environmental conditions, but even entire processes can switch to opposite functions, thus determining a different cell fate.

Given these premises, when we were invited as Guest Editors of a Special Issue, we had no doubts: to explore molecules and/or processes that behave like the two-faced god Janus.

This Special Issue, indeed, addresses this intriguing and relevant matter of research in molecular sciences. The landscape is complex since the dual behavior can be dependent on cell environment, genetic or epigenetic regulation, post-translational modifications and intricate interaction networks. How, though, can the same factor or process specifically exert opposite functions? When can it be considered as Dr. Jekyll or Mr. Hyde? Elucidating the molecular switches regulating this Janus behavior represents an interesting tool of investigation with important applications in the medical field.

As above reported, the Janus role is not restricted to molecules, but also regards cellular processes determining cell fate, such as the following: (1) signal transduction, the complex cell response to external inputs; (2) gene expression, the fine cell capability of encoding proteins or regulative RNAs; (3) autophagy, the form of intracellular material degradation that can represent a pro-survival response or culminate in cell death; (4) Cell stress response, the cell ability to respond to injury, toxicity, or oxidative stress; (5) Cell adhesion, the whole functional interactions that the cells establish with each other or with the extracellular matrix; (6) Cell transformation into cancer, the process that causes the cells to undergo uncontrolled proliferation, becoming tumor cells and acquiring malignancy.

This Issue aims to focus on these double-faced molecules/processes in the regulation of cell fate to provide a critical analysis of different situations and the regulative mechanisms involved in opposite cell responses.

The first contribution provided by Casanellas et al. [5] regards the Janus role of cell adhesion in differentiation. Specifically, the authors focus on chondrogenesis, which implies a continuous extracellular matrix remodeling to maintain healthy hyaline cartilage and minimize hypertrophy. Their results highlight the importance of controlling cell–substrate adhesion in the tissue engineering strategies for cartilage repair, which represent a goal in regenerative medicine.

Other contributions shed light on carcinogenesis and/or tumor progression. In this regard, Tsuchiya et al. elucidate the genomic mechanism that guides the cell-fate determination from embryo to cancer development [6]. They discuss how the genome resides in two states: a sub-critical state and super-critical one. The former maintains a certain perturbation at a local level whereas the latter allows a specific perturbation to spread over the entire system. In the case of a super-critical genome, the cell-fate can change. The authors provide a framework to develop a time-evolutional transition theory for the regulation of the cell-fate during carcinogenesis.

Cancer stem cells (CSCs) are widely considered as initiators of carcinogenesis [7]. The contribution of Kyriazi et al. specifically focuses on the dual effects of non-coding RNAs (ncRNAs) in cancer stem cell behavior [8]. The authors analyze the multifaceted contribution of microRNAs (miRNAs), long non-coding RNAs (lncRNAs) and circular RNAs (circRNAs), in sustaining CSC features and describe the molecular mechanisms of their action in different CSC types. They also propose a possible use of specific CSC ncRNAs as putative diagnostic and therapeutic biomarkers.

Strictly related with tumorigenesis and tumor progression is the hypoxia condition. Physiological oxygen concentration in tissues represents a hallmark of metabolic fitness and cellular wellness. On the other hand, the negative regulation of oxygen homeostasis accounts for cell damage and oncogenic transformation. The response to hypoxia implicates the activation of multiple genes, among which the hypoxia-inducible factor (HIF) plays a major role. Interestingly, increasing evidence suggests hypoxia signaling generates opposite responses in different cells and tissues [9]. In an interesting review, Corrado and Fontana widely discuss the dual and controversial role of hypoxia and the HIF-mediated pathway, with a particular focus on the immune system and cellular response to injury in both acute and chronic human diseases, including cancer, affecting different organs [10].

As regards the Janus roles referred to in proteins, the review of Tolomeo and Grimaudo on CCAAT/enhancer-binding proteins (C/EBPs) points out the ambiguous behavior of this protein family in carcinogenesis [11]. C/EBPs can be considered either tumor promoters or tumor suppressors, depending on the type of tumor, the isoform/s expressed in the cells, the type of dimerization (homo- or heterodimerization), the presence of inhibitory elements, and the ability to inhibit the expression of other tumor suppressors. The authors present a detailed description of each C/EBP family member, discussing their involvement in cancer and the opposite effects according to specific gene transcriptional activation.

Another key factor that can display an oncojanus behavior is p62, a versatile protein involved in the balance between cell death and survival. In our review, we describe the diversified roles of p62 through its multiple domains and interactors, and critically discuss the involvement of this protein in both cancer and neurodegeneration [12]. P62 is specifically involved in selective autophagy [13,14] and behaves as an interactive hub in multiple cell signaling. Chronic p62 accumulation is typical of many forms of tumors and stress granules present in neurodegenerative diseases, including lateral amyotrophic sclerosis, Parkinson disease and Alzheimer disease. On the other hand, the protein may also serve protective functions against tumorigenesis or neurodegeneration, thus displaying both oncojanus and neurojanus roles.

The pathway of the Wnt (Wingless and Integrated-1) family is one of the fundamental mechanisms that directs cellular proliferation and cell fate during embryonic development and tissue homeostasis. Consequently, mutations in this pathway are often linked to human birth defects, or different diseases, including cancer [15]. As discussed by Choi, the Wnt/β-catenin pathway also plays a role during the formation of new hair growth or hair loss [16]. Persistent hair loss represents a cause of psychological distress for many people worldwide. Therefore, elucidating the switch mechanisms regulating hair growth deserves particular attention. The authors focus on the role of Wnt/β-catenin in promoting hair growth, and describe a possible design for hair loss therapy by targeting the signal transduction affecting β-catenin.

Intriguingly, the HSP70 family proteins, which are known for their main role as chaperones during protein folding, also show a double behavior, like Dr Jekyll and Mr. Hyde, as reviewed by Tukaj [17]. They behave like Dr Jekyll when they protect the cells from cell stress conditions, but they can turn into Mr. Hyde when they exasperate the immune responses or promote cancer development. The author specifically focuses on the stress-inducible 70 kDa heat shock protein (Hsp70), highlighting its immunosuppressive activity and the importance of Hsp70 pharmacological induction in ameliorating autoimmune diseases. Moreover, the paper describes the effects of highly immunogenic extracellular Hsp70 in connection with immune responses and autoimmunity, considering the dual role of intra- and extracellular Hsp70 in the context of autoimmunity.

As expected, multitasking proteins are also widespread in the plant kingdom. Tian and Wang analyze the multiple roles played by Trasparent Testa Glabra1 (TTG1), a WD40 repeat protein [18]. TTG1 is involved in the regulation of different processes, including cell fate determination, secondary metabolisms, the accumulation of seed storage reserves, plant responses to biotic and abiotic stresses, and flowering time in plants. The different roles are dependent on the ability of TTG1 to work alone or to form complexes with different molecules, which determine the specific role.

Finally, in a perspective study, Duong et al. hypothesize a double antagonist role of RalGEFRGL-1, a protein involved in vulvar precursor cells’ fate patterning [19]. They speculate that RalGEFRGL-1 represents an “insulated switch”, whereby the promotion of one signaling activity reduces the promotion of the opposing activity. Therefore, this property most likely increases the impact of the switch on - fidelity more than two different proteins could do.

Overall, this issue does not pretend to be a complete description of the many aspects of the Janus behavior referred to in cellular processes and key proteins. An entire editorial series would not be sufficient to this aim. However, the major emphasis is on a review of different and even controversial cell molecules and processes, which are implicated in human diseases, including cancer, autoimmunity and neurodegeneration, or are important in regenerative medicine.

We thank all the authors for the time dedicated to providing these excellent contributions to this special issue of the journal. We also acknowledge the assistance of Lena Mao and the *IJMS* Editorial Office for their efforts in the preparation of this issue for publication.
